# MicroRNA-561-3p indirectly regulates the PD-L1 expression by targeting *ZEB1*, *HIF1A,* and *MYC* genes in breast cancer

**DOI:** 10.1038/s41598-024-56511-6

**Published:** 2024-03-10

**Authors:** Atena Yousefi, Fattah Sotoodehnejadnematalahi, Nahid Nafissi, Sirous Zeinali, Masoumeh Azizi

**Affiliations:** 1 Department of Biology, School of Basic Science, Science and Research Branch, Islamic Azad University, Tehran, Iran; 2 Breast Surgery Department, Iran University of Medical Sciences, Tehran, Iran; 3 Molecular Medicine Department, Biotechnology Research Center, Pasteur Institute of Iran, 69th Pasteur Street, Kargar Avenue, Tehran, Iran

**Keywords:** miRNA-561-3p, *ZEB1*, *HIF1A*, *MYC*, Breast cancer, Cancer, Molecular biology

## Abstract

Globally, breast cancer is the second most common cause of cancer-related deaths among women. In breast cancer, microRNAs (miRNAs) are essential for both the initiation and development of tumors. It has been suggested that the tumor suppressor microRNA-561-3p (miR-561-3p) is crucial in arresting the growth of cancer cells. Further research is necessary to fully understand the role and molecular mechanism of miR-561 in human BC. The aim of this study was to investigate the inhibitory effect of miR-561-3p on *ZEB1*, *HIF1A*, and *MYC* expression as oncogenes that have the most impact on PD-L1 overexpression and cellular processes such as proliferation, apoptosis, and cell cycle in breast cancer (BC) cell lines. The expression of *ZEB1*, *HIF1A*, and *MYC* genes and miR-561-3p were measured in BC clinical samples and cell lines via qRT-PCR. The luciferase assay, MTT, Annexin-PI staining, and cell cycle experiments were used to assess the effect of miR-561-3p on candidate gene expression, proliferation, apoptosis, and cell cycle progression. Flow cytometry was used to investigate the effects of miR-561 on PD-L1 suppression in the BC cell line. The luciferase assay showed that miRNA-561-3p targets the 3′-UTRs of *ZEB1*, *HIF1A* and *MYC* genes significantly. In BC tissues, the qRT-PCR results demonstrated that miR-561-3p expression was downregulated and the expression of *ZEB1*, *HIF1A* and *MYC* genes was up-regulated. It was shown that overexpression of miR-561-3p decreased PD-L1 expression and BC cell proliferation, and induced apoptosis and cell cycle arrest through downregulation of candidate oncogenes. Furthermore, inhibition of candidate genes by miR-561-3p reduced PD-L1 at both mRNA and protein levels. Our research investigated the impact of miR-561-3p on the expression of *ZEB1*, *HIF1A* and *MYC* in breast cancer cells for the first time. Our findings may help clarify the role of miR-561-3p in PD-L1 regulation and point to this miR as a potential biomarker and novel therapeutic target for cancer immunotherapy.

## Introduction

Breast cancer (BC) has been a significant threat to women's health. The incidence of BC is different in various ethnicities, and it is estimated that the number of BC cases will reach more than three million by 2050 worldwide. The mortality rate of BC in developed countries due to novel diagnostic and advanced medical treatment technology would be decreased^1,2^. Currently, BC treatments include surgery, radiotherapy, chemotherapy, and targeted therapy. Due to the heterogeneity of tumor tissue, the cancer stem cells (CSC) inside the tumor can self-renew and differentiate. Therefore, chemotherapy can lead to chemoresistance and the progression of the disease. It was shown that invasion and subsequent metastasis are the leading causes of death in about half of the BC patients^3,4^.

Programmed death ligand 1(PD-L1) is expressed by the CD274 gene in humans and could bind to PD-1 on T cells^5^. The PD-1/PD-L1 axis has a critical role in cancer cells escaping the immune surveillance^6,7^. Overexpression of PD-L1 in various human tumors suppresses the host immune system against cancer, and causes severe malignancies and poor prognosis^8,9^. PD-L1 is an immune checkpoint that has a crucial role in the strategies of BC therapy^9^. In BC, PD-L1 upregulation has been directly related to high-grade tumors, HER2-positive situations, negative estrogen receptor status, chemoresistance, and survival^10^.

In cancer cells, overexpression of PD-L1 occurs at different stages of gene expression regulation, including transcription, post-transcription and post-translational modifications, and exosomal transport. Therefore, increased PD-L1 expression suppresses the immune system's response to cancer cells, which causes the epithelial-to-mesenchymal transition (EMT) and cancer stem cell (CSC) phenotype, metastasis, and chemoresistance^11^.

EMT is related to the CSC phenotype^12^ and therefore, programmed EMT activation is a critical driver for the cancer stem cell phenotype during metastasis^13^. EMT regulation is done by different signaling pathways such as TGFβ^14^, Notch, and Wnt. On the other hand, it is affected by tumor environmental conditions such as hypoxia and the differential expression of microRNAs (miRs). In all of these pathways, common transcriptional f actors, including ZEB1, SNAI1, and MYC, are involved^15–17^.

ZEB1 ( zinc-finger E-box binding protein 1) encoded by the *ZEB1* gene (10p11.2) has a key role in the epithelial-to-mesenchymal transition network in BC^18,19^. Chen et al. demonstrated the critical role of the miR-200/ZEB1 axis in the regulation of PD-L1 expression either in the presence or absence of IFN-γ^20^. A high level of ZEB1 protein is observed in the tumorigenesis of all BC subtypes. It is worth mentioning that the ZEB1 promoter is a bivalent chromatin configuration, enabling the cancer stem cells to respond to some microenvironmental signals, such as TGF-β ^21^. ZEB1, as an EMT enhancer and miR-200 transcriptional agent, relieves miR-200 suppression of PD-L1 on tumor cells, resulting in immunosuppression of CD8^+^  T cells and metastasis^20^.

HIF1A (Hypoxia inducible factor 1A) is one of the influential external carcinogenic factors in the expression of PD-L1 and has a novel role in cancer target therapy such as immunotherapy^22–24^. In hypoxic environments, HIF1A promotes the drug resistance and metastasis of tumor cells. HIF1A acts as a major transcriptional driver in cancer progression^25^. HIF1A binds to specific regions called hypoxia response elements (HRE) on the PD-L1 promoter, promotes the expression of PD-L1, and reduces T-cell function, and results in immune suppression^26^ and stimulates EMT (epithelial-mesenchymal transition) in BC^27^. HIF1A overexpression, due to reduced glucose metabolism and increased lactate accumulation in hypoxic environments, creates a suitable condition around the tumor for both PD-1/PD-L1 interaction and immunosuppression of CD8+ T cells^28–30^.

The MYC oncogenic transcription factor is overexpressed in many cancer patients and regulates cell cycle, self-renewal, survival, cell growth, metabolism, protein synthesis, differentiation, and tumorigenesis^31,32^. MYC overexpression occurs through chromosomal translocation, genomic amplification, retroviral integration, and mutation in BC^33^. MYC overexpression is linked to promoting the expression of immune checkpoint gene products such as PD- L1^34^ at both the gene and protein levels in tumor cells^35^. MYC binds directly to the PD-L1 promoter region and, as a transcription factor, increases PD-L1 expression and reduces the antitumor immune response^34^.

MicroRNAs are small non-coding RNAs consisting of 20–22 nucleotides^36^ with diagnostic or therapeutic potential in cancer^37^. MicroRNAs are regulatory factors that regulate gene expression, cell differentiation and proliferation, tumorigenesis, and tumor progression^38^. Recent studies have shown that microRNAs can regulate PD-L1 expression, both directly and indirectly, by targeting the 3′-UTR of PD-L1 and related signaling pathway molecules, and hence have a significant role in BC therapy^39^.

Recently, miR-561 has been reported to be downregulated in gastric cancer^40^, NSCLC^41^, hepatocellular carcinoma^42^, and glioblastoma^43^ and promote cancer progression and metastasis (42 ), However, the function and molecular mechanism of miR-561 in human BC development remain unknown and need to be more investigated.

In the present study, the inhibitory effect of miR-561-3p on *ZEB1*, *HIF1A* and *MYC* expression was investigated. The mentioned oncogenes affect PDL-1 overexpression in BC cell lines, including MCF-7 (less aggressive BC), MDA‐MB‐231, and BT-549 (metastatic BC model). Moreover, the effects of miR-561-3p overexpression on proliferation, apoptosis, and G1 arrest through downregulation of candidate oncogenes in BC cell lines were investigated.

## Results

### MiR-561-3p expression was down-regulated and *MYC*, *HIF1A*, and *ZEB1* expression were up-regulated in BC tissues

**Figure 1:**
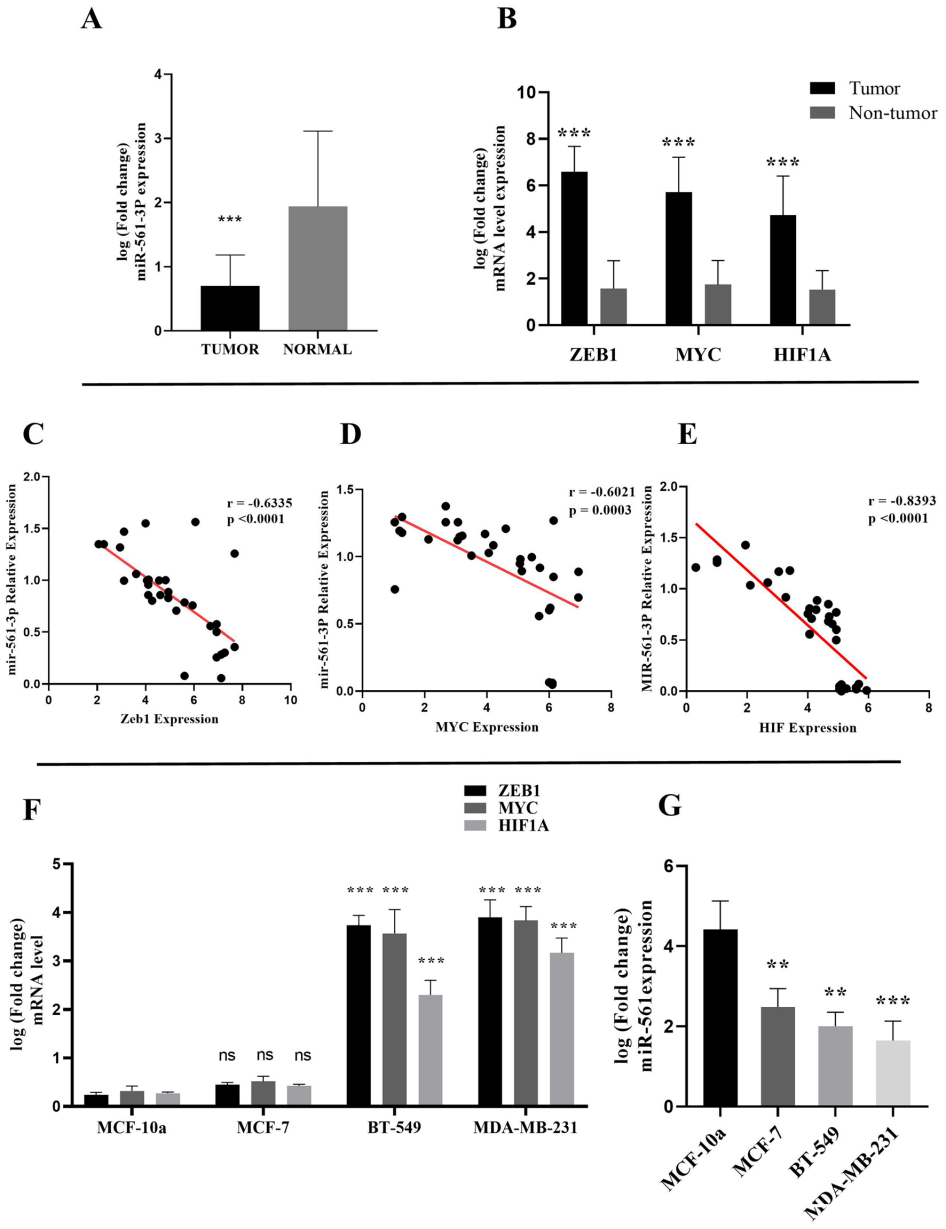
miR-561-3p expression was correlated with the *ZEB1, MYC,* and* HIF1A* expression in BC tissues and cell lines. (**A**) qRT-PCR analysis of miR-561-3p and (**B**) *ZEB1, MYC*, and *HIF1A* expression in 45 paired BC tissues and corresponding adjacent normal tissues. Correlation analysis between the relative expression level of (**C**) ZEB1, (**D**) MYC, and (**E**) HIF1A, and miR‐561-3p expression in human BC tissues. (**F,G**) qRT-PCR analysis of miR-561-3p and ZEB1, MYC, and HIF1A expression in three BC cell lines (MCF-7, MDA-MB-231, and BT-549) and human normal breast epithelial cell line (MCF-10A) as a control. ***P < 0.001, **P < 0.01, *ns* non-significant.

The expression levels of miR-561-3p and MYC, HIF1A, and ZEB1 genes in 45 pairs of PD-L1-positive BC tissues and their adjacent normal tissues were initially examined PD-L1- positive tissues obtained from Hajibabaei et al.^44^. The results of qRT-PCR revealed that the expression levels of miR-561-3p were abnormally down-regulated (1.235 ± 0.189) in BC samples compared with paired healthy breast tissue samples (Fig. 1A, P < 0.001). Moreover, the results showed that the expression of *ZEB1*, *MYC*, and *HIF1A* genes was significantly increased (5.025 ± 0.26, 3.970 ± 0.25, 3.191 ± 0.24 respectively) in BC tissues compared with adjacent normal tissues (Fig. 1B, P < 0.001). Pearson correlation analyses of *ZEB1*, *MYC*, and *HIF1A* gene expression in BC tissues demonstrated a negative correlation between miR‐561-3p expression and *ZEB1* (Fig. 1C, r = −0.6335; P < 0.0001), *MYC* (Fig. 1D, r = −0.6021; P = 0.0003), and *HIF1A* mRNA levels(Fig. 1E, r = −0.8393; P < 0.0001). Furthermore, a negative relationship was observed between the expression of *ZEB1*, *MYC*, and *HIF1A* genes and miR‐561-3p expression in BC cell lines. In the BC cell lines, the *ZEB1*, *MYC*, and *HIF1A* genes were upregulated (Fig. 1F, P < 0.001) but the miR‐561-3p was downregulated compared to the MCF-10a cell line(Fig. 1G, P < 0.01, P < 0.0010). The results showed that the expression of PD-L1 gene was significantly increased in BC tissues compared with adjacent normal tissues (Supp. 1A, P < 0.001). Pearson correlation analyses of *ZEB1*, *MYC*, and *HIF1A* gene expression in BC tissues demonstrated a positive correlation between *PD-L1* expression and *ZEB*1 (Supp. 1B, r = 0.5098; P = 0.0003), *MYC* (Supp.1C, r = 0.5824; P < 0.0001), and *HIF1A* mRNA levels (Supp. 1D, r = 0.4037; P < 0.0066). The results of qRT-PCR revealed that the expression levels of ZEB1 (Supp. 1E, P < 0.001), *MYC* (Supp.1F, P < 0.001), and *HIF1A* (Supp. 1G, P < 0.001), *PD-L1* genes (Supp.1H, P < 0.001) were significantly increased and miR-561-3p (Supp. 1I, P < 0.001, P < 0.05) was abnormally down-regulated in BC tissues stage III compared with BC tissues stage I.

### Bioinformatic tools and luciferase reporter assay confirmed the 3′-UTRs of *MYC*, *HIF1A*, and *ZEB1* genes as targets of miR-561-3p

**Figure 2:**
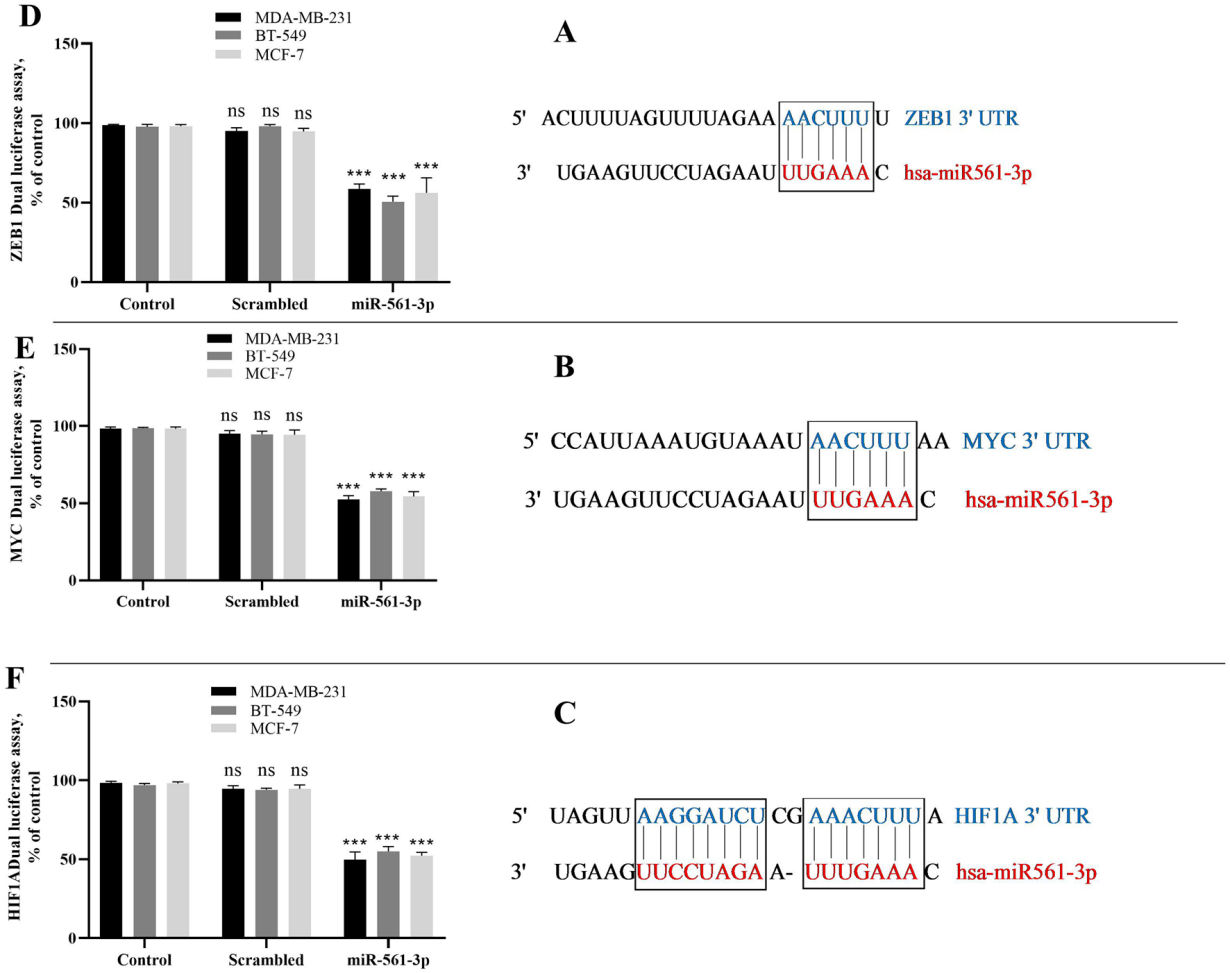
miR-561-3p directly targets ZEB1, MYC, and HIF1A genes. The 3′-UTR_s_ sequence of the (**A**) ZEB1, (**B**) MYC, and (**C**) HIF1A ‘genes and the miR-561-3p binding sites. Prediction of the target genes for miR-561-3p using an online miR target prediction database. (**D**) MDA-MB-231, (**E**) BT-549, (**F**) MCF-7 cells were transfected with the recombinant psiCHECK^TM^-2 vector harboring the ZEB1, MYC, and HIF1A 3′-UTRs, and vectors cloned by miR-561-3p or scrambled microRNA. Dual-Luciferase assays were performed for each cell line and decrease in relative Renilla luciferase activity was observed, but relative Renilla luciferase activity was not significantly changed in the cells treated with the scrambled microRNA. ***P < 0.001, *ns* non-significant, n = 3. Data represent as mean ± SE from three independent performed experiments.

In this study, the 3′-UTRs of *ZEB1*, *MYC*, and *HIF1A* genes was analyzed as potential target sites for miR-561-3p. Online databases such as TargetScan 4.0^40^ were used to predict the candidate targets. According to the prediction tools, the 3̀-UTRs of *ZEB1*, *MYC*, and *HIF1A* genes have been considered as putative miR-561-3p binding sites (Fig. 2A–C). For in vitro confirmation, the *ZEB1*, *MYC*, and *HIF1A* 3̀-UTR sites and scrambled sequence were cloned into the downstream Renilla luciferase gene in psiCHECK-2 vector. These plasmids were cotransfected with miR-561-3p and scrambled microRNA into MDA-MB-231, BT-549, and MCF-7 cells. The results show that miR-561-3p significantly reduced the relative luciferase activity of the *ZEB1*, *MYC*, and *HIF1A* 3̀-UTR plasmids compared with the controls. Transfecting miR-561-3p into MDA-MB-231, BT-549, and MCF-7 cells harboring *ZEB1* 3̀-UTR decreased the luciferase activity to 39.8 ± 1.43%, 47.2 ± 1.45%, and 42 ± 4.90%, respectively (Fig. 2D) while the luciferase activity for *MYC* decreased to 45.9 ± 0.78%, 41 ± 0.54%, and 43.89 ± 1.09%, respectively (Fig. 2E). The results showed that the luciferase activity for *HIF1A* decreased to 47.98 ± 2.24%, 42  ±  1.15%, and 44.89 ± 0.62%, respectively (Fig. 2F) as compared with control (P < 0.001) The results confirmed the *MYC*, *ZEB1*, and *HIF1A* genes as candidate targets for miR-561-3p, and also these 3′-UTRs as functional target sites for this microRNA in BC cells.

### MiR-561-3p directly downregulate the expressions of *MYC*, *HIF1A*, and *ZEB1* genes in BC cells

**Figure 3:**
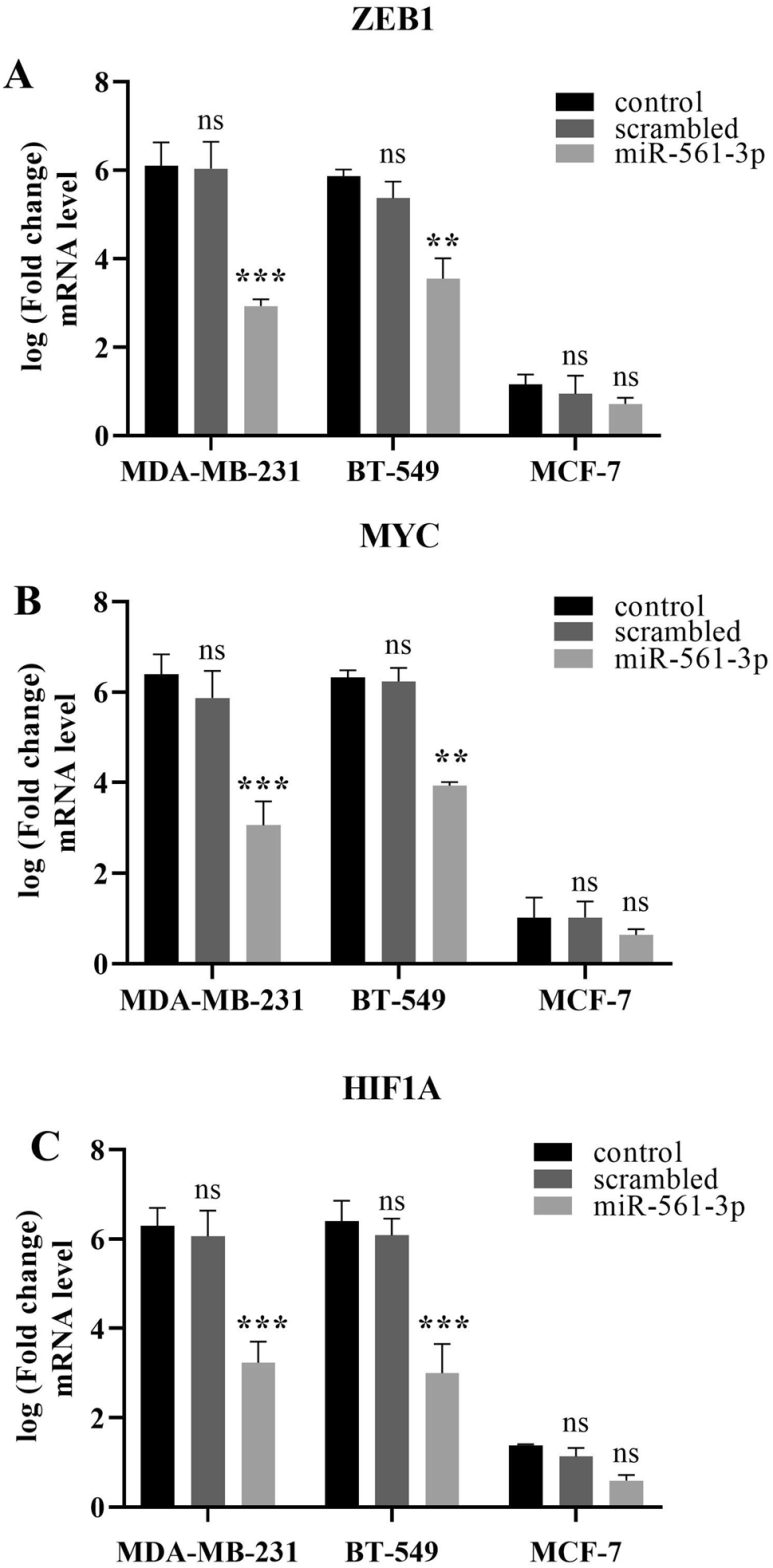
miR-561-3pdownregulated the MYC, HIF1A, and ZEB1 mRNA in BC cells. MDA-MB-231, BT-549, and MCF-7 cell lines were transfected with miR-561-3p or scrambled microRNA. After 48 h, ZEB1(**A**), MYC (**B**), and HIF1A (**C**) expression were evaluated by real-time quantitative PCR. The decrease in relative mRNAs expression was evident in cells treated with miR-561-3p, while no changes were observed with the scrambled microRNA, ***P < 0.001, **P < 0.01, *ns*  non-significant, n = 3. Data represent as mean ± SE from three independent performed experiments.

To confirm the hypothesis that overexpression of miR-561-3p could downregulate the expression of *ZEB1*, *MYC*, and *HIF1A* genes in BC cell lines, the miR-561-3p or scrambled oligonucleotide was transfected into MDA-MB-231, BT-549, and MCF-7 cells, and the level of MYC, HIF1A, and ZEB1 mRNA was measured by quantitative real-time PCR (Fig. 3, P < 0.01). The results showed that the overexpression of miR-561-3p is followed by a reduction in the level of ZEB1, MYC, and HIF1A mRNA in MDA-MB-231, BT-549, and MCF-7 cell line (Fig. 3A–C) (mean ± SE) compared to non-transfected cells.

### MiR-561-3p indirectly downregulates the PDL-1 in BC cells

**Figure 4:**
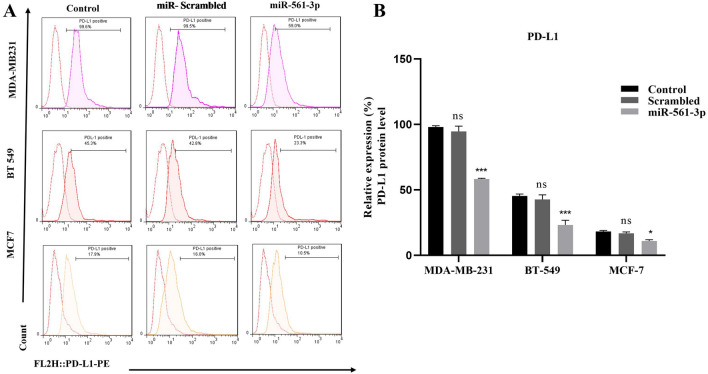
miR-561-3p indirectly suppresses the expression of PD-L1 in BC cell lines. miR-561-3p overexpression decreased MDA-MB-231, BT-549, and MCF-7 cell-surface PD-L1 protein. (**A**) FACS analysis of PD‑L1 in breast cancer cell lines. Anti-human-PD-L1 was used as a binding antibody to cell-surface PD-L1 protein. (**B**) Graphs representing PD‐L1 protein expression levels in MDA-MB-231, BT-549 and MCF-7 cell lines, *P < 0.05. *** P < 0.001, *ns* = non-significant, n = 3. Data represent as mean ± SE from three independent performed experiments.

In this study, miR-561-3p or scrambled oligonucleotide was transfected into different BC cell lines (MDA-MB-231, BT-549 and, MCF-7 cells), and the cell surface expression of PD-L1 protein was measured on live cells by flow cytometry. As shown in (Fig. 4A, B), the overexpression of miR-561-3p significantly downregulated PD-L1 expression in MDA-MB-231 (41% ± 0.24%0, BT-549 (22.1% ± 1.14%), and MCF-7 (7.9% ± 0.19%), Additionally, we showed that overexpression of miR-561-3p indirectly downregulated PD-L1 at protein levels compared with non-transfected cells (P < 0.05, P < 0.001).

### MiR-561-3p inhibited cell proliferation and induced apoptosis in BC cell lines

**Figure 5:**
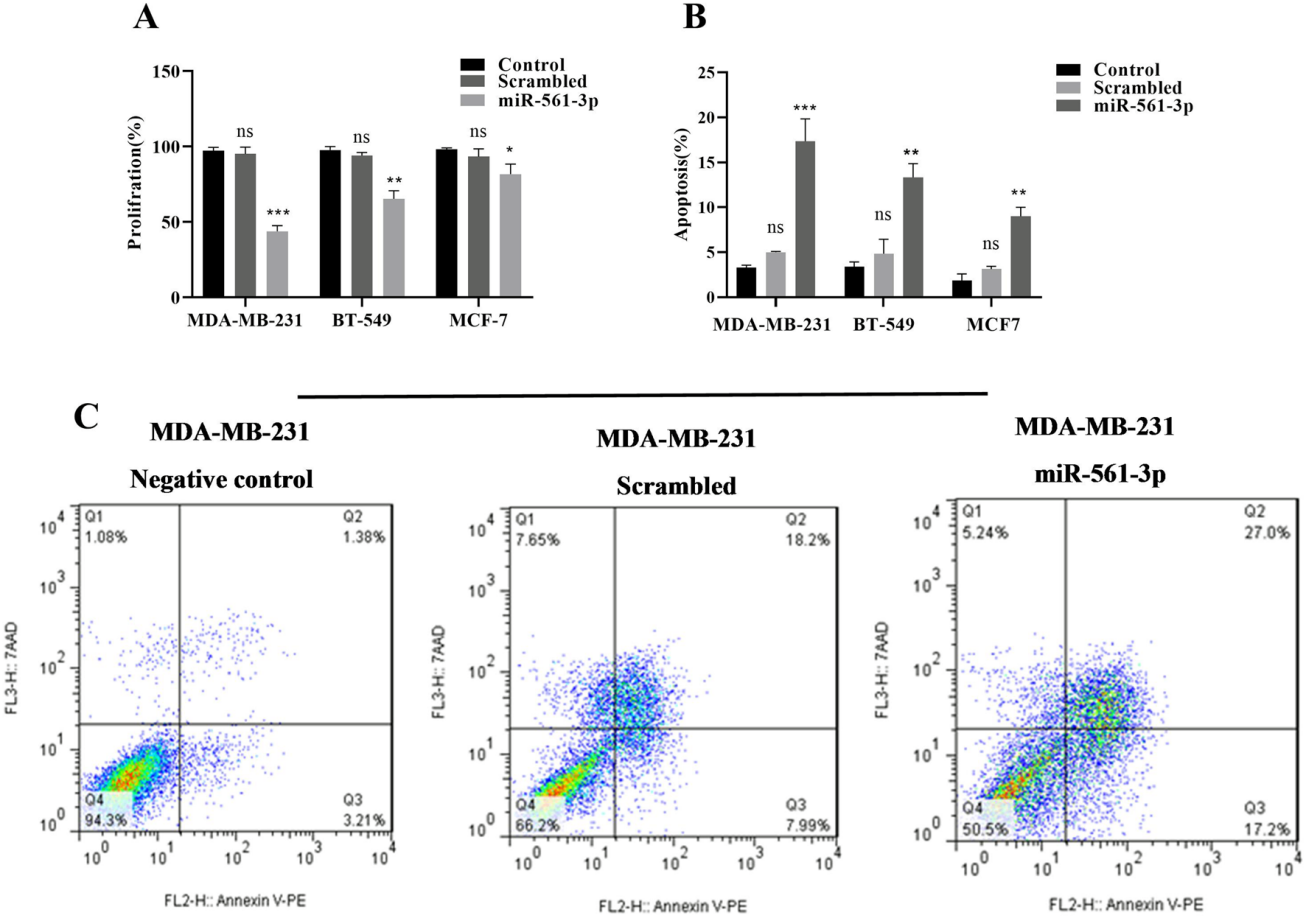
Upregulation of miR-561-3p suppresses BC cell lines proliferation and increases apoptosis. (**A**) The rate of MDA-MB-231, BT-549, and MCF-7 cells proliferation is decreased after transfection with miR-561-3p. The results showed the ratio of cell proliferation after miR-561-3p overexpression. *P < 0.05, ***P < 0.001, n = 3. (**B**) The rate of MDA-MB-231, BT-549, and MCF-7 cells apoptosis is increased after transfection with miR-561-3p. *P < 0.05, ***P < 0.001, *ns * non-significant, n = 3. (**C**) Representative histograms depicting apoptosis of MDA-MB-231 cells transfected with miR-561-3p or scrambled microRNA and nontransfected cells. Data represent as mean ± SE from three independent performed experiments.

The inhibitory role of miR-561-3p on BC cell lines, including MDA-MB-231, BT-549, and MCF-7, following transfection with miR-561-3p was evaluated using an MTT assay. The results of the MTT assay revealed that overexpression of miR-561-3p significantly suppressed the proliferation of BC cell lines after 72 h of transfection (Fig. 5A). The proliferation of MDA-MB-231, BT-549, and MCF-7 cells was significantly decreased to 53.5% ± 0.96%, 32.4% ± 1.78, and 16.2% ± 3.26, respectively compared with the control group (non-transfected cells and scrambled miR-transfected cells).

The apoptosis induction effect of miR-561-3p on the BC cells was evaluated 48 h after transfection using Annexin-PI staining. As shown in Fig. 5B, C, the overexpression of miR-561-3p significantly induced apoptosis in MDA-MB-231 (14.06% ± 1.27%), BT-549 (9.93% ± 0.57%), and MCF-7 (7.16 ± 0.44(% cell lines compared with control microRNA (P < 0.05, P < 0.01, P < 0.001). These results suggested that overexpression of miR-561-3p by targeting *ZEB1*, *MYC*, and *HIF1A* genes can induce apoptosis and reduce cell proliferation in BC cell lines. 

### MiR-561-3p arrested the cell cycle

**Figure 6:**
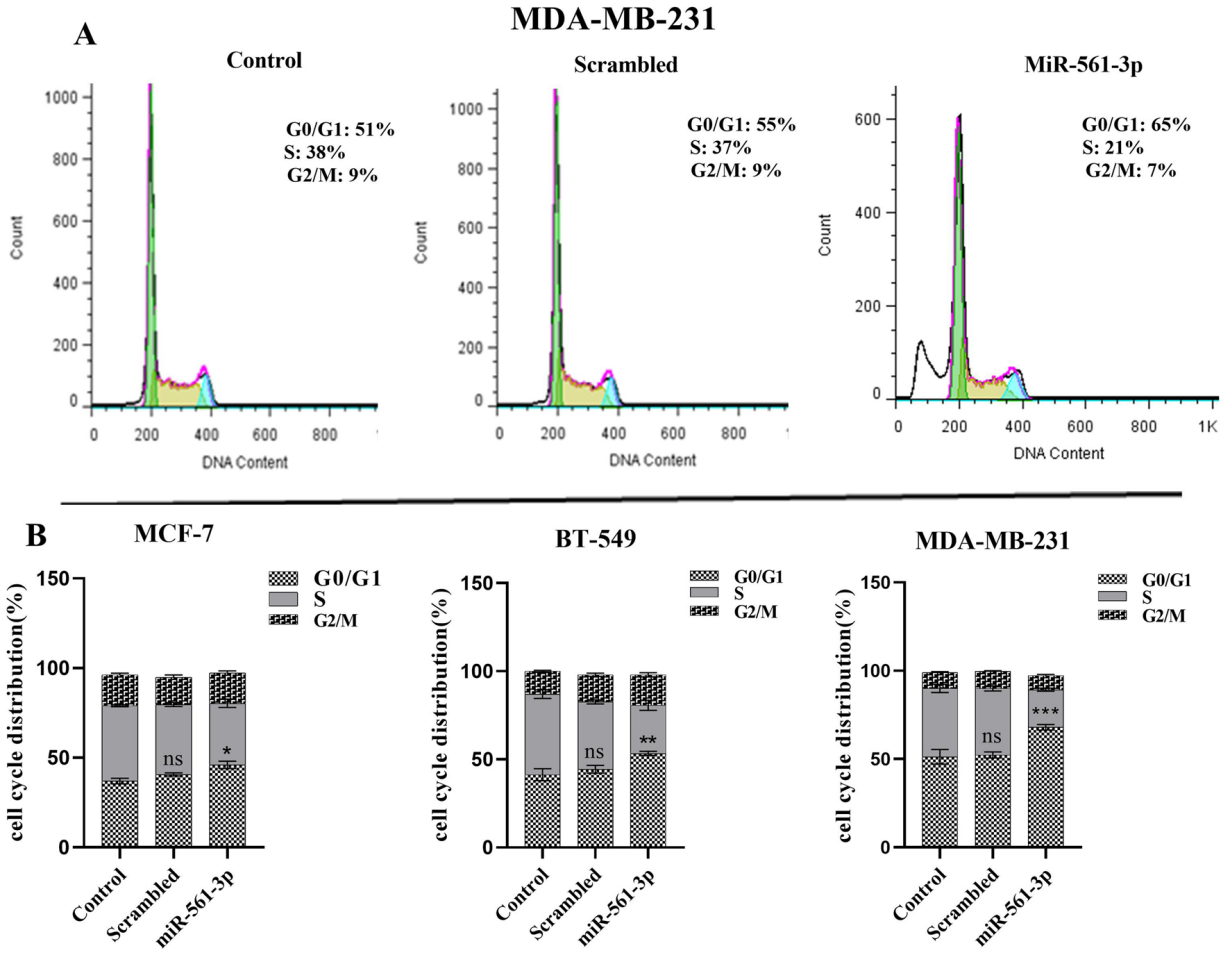
Upregulation of miR-561-3p suppresses BC cell lines proliferation and induces G0/G1 and decrease G2/M cell cycle arrest. (**A**) The histogram represents the proportion of cells in the G0/G1, S, and G2/M phases after miR-561-3p overexpression in MAD-MB-231. (**B**) The ratio of MDA-MB-231, BT-549, MCF-7 cells in the G0/G1, S, and G2/M phases 48 h after miR-561-3p transfection was illustrated. *P < 0.05, **P < 0.01, ***P < 0.001, *ns*  non-significant, n = 3. Data represent as mean ± SE from three independent performed experiments.

A flow cytometry assay was used to evaluate the cell cycle arrest induced by miR-561-3p in MDA-MB-231, BT-549, and MCF-7 cell lines after 48 h of transfection. The results demonstrated that overexpression of miR-561-3p increased the G0/G1 phase fraction (P < 0.05) and decreased the S and G2/M phase fractions ) P < 0.05) (Fig. 6A, B).

## Discussion

In BC, the bulk of malignant tumors are associated with the number of tumor-infiltrating lymphocytes (TILs) or immune gene expression signatures^45,46^. The malignant cells and somatic mutations that cause immunogenic death are associated with inferior outcomes and chemotherapy resistance; thus, an effective immune system is critical in producing neoantigens that elicit an adaptive immune response and will clear or keep the escaping tumor cells dormant^47^. Therefore, identifying molecular pathways involved in the development and progression of BC is crucial for the investigation of novel and practical diagnosis markers, as well as potential therapeutic targets, which are urgently needed to understand^48^. In the last few years, a new BC treatment based on ICI (immune checkpoint inhibitors) such as anti-PD-1/PD-L1 agents is a promising strategy. Better response to ICI (immune checkpoint inhibitor ) treatment may be predicted by PD-L1-positive status, non-liver metastases, first-line immunotherapy, and high TIL and CD8 + T-cell infiltration levels. Patients with PD-L1-positive tumor could gain more survival benefits from immune checkpoint therapy^49^. However, single-cell transcriptomic sequencing of breast cancer cells that metastasized to the liver and brain showed that the expression of the immunoreceptor inhibitory checkpoint genes, LAG3 and TIGIT, in T cells was higher than that of PDCD1 (PD-1). Based on these observations, individuals with liver and brain metastasis may benefit from therapeutic targeting of these two second-generation immune checkpoint receptor targets and their ligands (LGALS3 and NECTIN2)^50^.

Sabatier et al. analyzed 5454 BCs DNA microarrays and PD-L1 mRNA of 45 BC cell lines, and their finding showed PD-L1 overexpression in 20% of clinical samples and 38% of basal tumors, and they suggested that PD-L1 overexpression was related to decreased T cell cytotoxic immune response^51^. Muenst et al*.* assayed PD-L1 expression in 650 evaluable formalin-fixed BC sample cases, and they reported that 152 (23.4%) of the 650 BC specimens were PD-L1 positive^52^.

In March 2019, antibodies targeting PD-1/PD-L1, such as atezolizumab plus nab-paclitaxel, were approved by the Food and Drug Administration (FDA) to increase the survival rate in advanced TNBC patients with PD-L1-positive tumors. Soon, anti-PD-1/PD-L1 agents are going to make impressive achievements in BC therapy. However, combination therapeutic strategies, including immune checkpoint blockade and multi-agent chemotherapy to increase patients' survival rates, are often administered in the early and advanced stages^53,54^.

Recently, studies have suggested that disregulation of miRs plays vital roles in the initiation and progression of BC via targeting PD-L1 expression^2–4^. For example, Dastmalchi et al*.* reported that miR-424-5p inhibited proliferation and invasion of BC cells through targeting PD-L1 and modulating the PTEN/PI3K/AKT/mTOR signaling pathway; therefore, miR-424-5p is considered a tumor-suppressor miR in BC^2^. LanlanGao et al*.* showed that miR-873 could reduce the stemness and chemoresistance of BC cells by targeting PD-L1 expression, which eventually suppressed the PI3K/Akt and ERK1/2 pathways^4^. Azarbarzin et al*.* demonstrated that inhibition of cell proliferation and migration and apoptosis induction could happen in BC cells via miR-383-5p transfection. MiR-383-5p has a role as a tumor suppressor through an inhibitory effect on the PI3K/AKT/mTOR pathway and inhibiting tumor PD-L1 expression^55^. According to the study of Rasoolnezhad et al., miR-138–5p could induce apoptosis and cell cycle arrest, and inhibit migration and proliferation via targeting PD-L1 and then, PI3K/AKT pathway in BC^56^. In another study, it was showed that the 3̀-UTR of both PD-L1 and LDHA genes have binding sites for miR-34a. These acted as ceRNAs (competitive endogenous RNAs) to promote the expression and function of each other through regulation of miR-34a in TNBC^57^. In this study, we investigated the effect of miR-561-3p on proliferation, apoptosis, and cell cycle arrest via indirect targeting of PD-L1 in BC.

Some studies have reported that miR-561-3p plays a crucial role in cancer progression. For example, Kun Qian et al*.* have studied the potential role of miR-561-3p in gastric cancer development, and their finding confirmed that miR-561-3p inhibits cell proliferation and invasion by downregulating c-MYC expression^40^. Karimi et al*.* demonstrated the inhibitory effect of miR-561-3p on glioblastoma cell proliferation and apoptosis via targeting c-MYC expression. This finding suggests that miR-561-3p is a promising agent for GBM (glioblastoma multiforme) treatment^43^. Liao et al*.* demonstrated that miR-561-3p has an inhibitory role on cell cycle and proliferation by targeting P-REX2a and then, regulating the PTEN/AKT signaling pathway in NSCLC^41^.

Currently, the effect of miR-561 has not yet been investigated in breast cancer. Here, for the first time, we investigated the functionality of this miR in the inhibition of breast cancer. In an unpublished study, Hajibabaei et al*.* found that MALAT1 knockdown by siRNA could prevent BC cell progression through the miR-561-3p/TOP2A axis^58^. PD-L1 is regulated at the genomic, epigenetic, transcriptional, and post-translational levels^59^. In another study conducted by Hajibabaei et al*.* the direct inhibition of PD-L1 by miR-145 and miR-335 was discussed in breast cancer^44^ and, in this study, miR-561-3p was used to indirectly target the *PD-L1* gene via *ZEB1*, *MYC*, and *HIF1A* genes. First, we selected PD-L1-positive BC tissues and examined the expression of miR-561-3p in these specimens. We found that miR-561-3p was downregulated in tumor samples compared to normal, As we mentioned above, over expression of MALAT1 can be the probable reason for the downregulation of miR-561-3p in BC^58^. Using bioinformatics analysis, we demonstrated that *ZEB1*, *MYC*, and *HIF1A* have potential as novel targets for miR-561-3p. The dual-luciferase reporter assay confirmed that miR-561-3p could directly target the *ZEB1*, *MYC*, and *HIF1A *3′-UTRs and inhibit their transcription. In the current study, the qRT-PCR results showed an opposite relationship between ZEB1, HIF1A, and MYC mRNA expression and miR-561-3p overexpression in BC tissues. Based on these results, miR-561-3p may play an important role as a tumor suppressor in BC.

In various types of human cancer, including colorectal^60^, cervical^61^, gastric^62^, and bladder cancer^63^, ZEB1 has been upregulated, and its overexpression has some effects on cellular processes, including cell growth, apoptosis, migration, invasion, metastasis, tumor development, and tumor progression^64,65^. In TNBCs (triple-negative BCs) and basal-like BCs, the level of ZEB1 is higher than the luminal subtype^66^. Moreover, ZEB1 overexpression has a role in resistance in therapy, poor survival, and increased metastatic risk in BC^66^.

The ZEB1 promoter is activated by the TGF-β signaling pathway (21 ). Therefore, the transition of cell phenotypes from CD44-low to CD44-high is increased, and then the growing generation of CSC signals occurs in BC^21^. Ataxia-telangiectasia mutated (ATM) kinase phosphorylates ZEB1 and leads to growing ZEB1 stabilization in response to DDR (DNA damage repair)^67^. In BC patients with ZEB1 overexpression, chemoresistance via inadequate responses to EPI (epirubicin) was observed^68^. The double-negative feedback loop between ZEB1 and the miR-200 axis plays a critical role in regulation of PD-L1 overexpression. The miR-200c expression can be inhibited in cancer cells by aberrant expression of EMT-factors, such as ZEB1^69^, and consequently, the expression of PD-L1 (as a target of miR-200 increased )^20^. By creating EMT phenotypes and stem cell-like features in tumor cells, ZEB1 can play an important role in the development of BC. Therefore, inhibition of ZEB1 expression is a worthy suggestion for the treatment of BC^68,70^.

ZEB1 was a target of miR-601 and miR-448, and overexpression of this gene could reverse the suppressive effects of miR-601 and miR-448 on the development of BC^71,72^. It was showed that miR-409-3p targets ZEB1 to control the invasion and metastatic process of BC. This suggests that miR-409-3p could be a novel prognostic marker and therapeutic target for the treatment of BC metastasis^73^.

HIF1A can bind to HRE regions of the PD-L1 promoter and promotes the expression of PD-L1^26^. Meanwhile, inhibiting HIF1A signaling could reduce PD-L1 expression in multiple types of cancers^26,74^ HIF1A activation promotes glycolytic metabolism, angiogenesis, and carcinogenesis^75^. Furthermore, HIF1A can overexpress LDH-A (lactate dehydrogenase) which thus facilitates the accumulation of lactate in the tumor microenvironment and promotes tumor invasiveness, drug resistance, and immune escape of tumor cells^76^. Excessive activity of hypoxia-related pathways is correlated with the proliferation ability of CTCs (circulating tumor cells) in the brain and also blood-borne metastasis to the brain in women with advanced metastatic BC^77^, Therefore, HIF1A targeting could be a promising therapeutic approach for cancer.

Under hypoxic conditions, HIF-1A was upregulated in breast cancer cells, and overexpression of some miRNAs such as miR-497 and miR-7641 can suppress this gene^78,79^. In a nude mouse xenograft tumor model, miR-497 suppressed tumor growth and decreased angiogenesis^78^. Down-regulating HIF-1A expression in breast cancer cells may be one of the mechanisms by which miR-7641 suppresses cancer stemness^79^.

MYC is a proto-oncogene in many cancers, including Burkitt's lymphomas, lung carcinoma, breast carcinoma, and colon carcinoma. Moreover, MYC, as a proto-oncogene, can regulate cell differentiation, proliferation, and apoptosis^80^. Amplification of MYC is present in 30–50% of high-grade BCs and is related to resistance in anti-cancer therapies; therefore, it is a useful predictive marker for drug and RFS (relapse-free survival (^81^. MYC binds directly to the PD-L1 promoter region and, as a transcription factor, increases PD-L1 expression. MYC participates in PD-L1 regulation,interacts with BC stem cell markers such as CD44, CD24, and ALDH1, and plays a significant role in the regulation of the initiation and metastasis of BC^80^. MYC overexpression inhibits BRCA1’s tumor suppressor and causes the development of basal-like BC; therefore, targeting MYC-regulated pathways provides a promising therapeutic strategy for BC^82^.

The other study, examined the c-myc/miR-29b-3p/CDK6 axis, is thought to be responsible for downregulating miR-29b-3p by c-myc to enhance CDK6 activation and induce palbociclib resistance in breast cancer. This was done using both xenograft models and patient-derived tumor xenograft (PDTX) models. They proposed c-myc as a potential biomarker for breast cancer patients' susceptibility to palbociclib. Kinase inhibitors, such as palbociclib, aid in preventing or postponing the spread of cancer cells^83^.

In this study, the ability of miR-561-3p to decrease proliferation and induce apoptosis and cell cycle arrest was examined by transfecting miR-561-3p into MDA-MB-231, BT-549, and MCF-7 cell lines. The results demonstrated that miR-561-3p could downregulat the expression of *ZEB1, HIF1A,* and *MYC* genes. In this study, we examined the effects of miR-561-3p overexpression in BC in two ways. First, miR-561-3p could decrease the expression of PD-L1 by targeting the *ZEB1, HIF1A,* and *MYC* genes. Second, downregulation of PD-L1 by targeting some signaling pathways such as MAPK and PI3K/Akt.Also, targeting some transcriptional factors that are well known to reduce cancer development through boosting the immune response against cancer cells, inhibiting cell proliferation, inducing apoptosis^84^, and arresting the cell cycle^85^. On the other hand, downregulation of *ZEB1, HIF1A,* and *MYC* genes could prevent the development of BC by inhibiting cell proliferation and increasing apoptosis^20,64,65,75,80^.

The results of this study demonstrated that miR-561-3p may indirectly affect PD-L1 expression by degrading ZEB1, HIF1A, and MYC mRNA, stopping BC cell proliferation, and inducing apoptosis. Based on our results, targeting ZEB1, HIF1A, and MYC may be a novel strategy for BC therapy, and miR-561-3p is a novel and promising candidate for microRNA restoration therapy in BC patients. To attain more accurate outcomes for the analysis of the manifestation of the PD-L1 gene and its impact on the progression of BC, it is necessary to have more sample volumes. Moreover, it could be better to perform some supplementary tests, such as RNA sequencing for deep knowledge of the role of PD-L1, ZEB1, MYC, HIF1A gene expression in BC.

## Materials and methods

### BC Cell lines and tissue samples

Human BC cell lines, including MCF‐7, MDA‐MB‐231, and BT-549 were purchased from the Iran National Cell Bank (Pasteur Institute of Iran, Tehran). MCF‐7 and MDA‐MB‐231 cell lines were propagated in Dulbecco's modified Eagle medium (DMEM), and BT-549 cell line was cultured in Dulbecco's modified Eagle medium (DMEM-high glucose) (Gibco, Germany). Both DMEM and DMEM-high glucose media were supplemented with 10% fetal bovine serum (FBS) (bio sera, South America), streptomycin (100 μg/ml), and penicillin (100 U/ml). The cells were incubated at 37 °C with 5% CO_2_ and 80% humidity. Forty five human BC tissue samples and adjacent normal tissues were obtained from khatamol Anbia Hospital and instantly frozen in liquid nitrogen, and stored in −70 °C. Clinical data of the patients, including sex, age, tumor differentiation, lymph node metastasis, and clinical data were obtained from the hospital records (Table 1).

**Table 1:** Demographic and clinical characteristics of the breast cancer patients.

Characteristics	N (45)
Age	
> 45	28
< 45	17
Estrogen receptor status	
ER+	16
ER−	29
Progesterone receptor status	
PR+	20
PR−	25
Her2/neu receptor status	
Her2/neu+	21
Her2/neu−	24
Histologic subtype	
Ductal	19
Lobular	22
Other	4
Lymph node involvement	
Negative	20
Positive	25
Stage	
I	3
II	9
III	33
Ki67	
0–30%	13
30–60%	32

### RNA extraction, reverse transcription, and RT‐qPCR

Total RNA of BC cell lines, normal tissues, and tumor samples was extracted with QIAzol^®^ (Qiagen, Germany). cDNAs were synthesized by M-MuLV reverse transcriptase enzyme included in the cDNA synthesis kit (Fermentas, EP0351) according to the manufacturer’s protocol. GAPDH was used as a housekeeping and normalizer for all target mRNAs. Each qPCR assay was performed in triplicate. RT‐qPCR reactions were prepared in a final volume of 25μl containing 140ng of specific primers (Table 2) and 12.5μL SYBR green master mix (Life Technology, 4309155). RT‐qPCR reaction was performed by the following program: enzyme activation at 95 °C for 10 min, followed by 40 cycles of denaturation at 95°C for 20 s, annealing at 59 °C for 30 s, and extension at 72 °C for 30 s. Statistical analysis for relative mRNA expression was performed by the Relative Expression Software Tool (REST) proposed by Pfaffl^30^. Fold change and P-value (< 0.05) were determined by REST, which considered all software requirements. All PCR assays displayed efficiencies between 1.8 and 2.0. Primers for real-time PCR were designed by Oligo version 7.56 and check them for specificity by using Primer-BLAST software (http://blast.ncbi.nlm.nih.gov/9 ((Table 2). All oligos were purchased from Cinaclon Company(Iran,Tehran).

**Table 2:** Primer sequence for real-time PCR analysis.

Gene	Sequence of oligonucelotides from 5′to 3′for real-time PCR analysis
ZEB1	Forward: TACCAGAGGATGACCTGCCA Reverse: TGCCCTTCCTTTCCTGTGTC
HIF1A	Forward: GAGAGGTTGAGGGACGGAG Reverse: GAGACTTTTCTTTTCGACGTTCA
MYC	Forward: GTAGTCGAAAACCAGCCTCCC Reverse: TTCTCCTCCTCGTCGCAGTA
miR-561-3p	Forward: CGCTCCAAAGTTTAAGATCCTTGAAG Reverse: AGACTGCACCTGTCCGG
miR-561-stemloop	GTCGTATCCAGTGCAGGGTCCGAGGTATTCGCACTGGATACGACACTTCA
U6	Forward: CTCGCTTCGGCAGCACA Reverse: AACGCTTCACGAATTTGCGT
GAPDH	Forward: ACACCCGCTCATCAATCTTT Reverse: AGGTCCACGACTCTGTTGCT

### Designing the stem-loop

The miR-561-3p sequence was obtained from the miRbase website (http://www.miRbase.org/). To design the stem-loop structure with increased flexibility and necessary sensitivity, the sequence of Chen et al*.* was modified. Also, the loop was enlarged to design a universal reverse primer and TaqMan probe inside it and nucleotide replacement was performed to decrease the melting temperature of the stem part. All of these modifications added 14 nucleotides to the original sequence of miR-561-3p (Table2). In order to detect miR-561-3p in the BC normal and tumor tissues by real-time PCR, the complete sequence of miR-561-3p as the forward primer was used, and also U6 as the reference gene. All oligonucleotides were purchased from the Cinaclon Company (Iran, Tehran).

### Bioinformatics’ prediction

The miR-561-3p sequence was obtained from the MiRbase website (http://www.miRbase.org). Binding of miR-561-3p to the 3′-UTRs of ZEB1 (NM_001174096.1), MYC (NM001354870.1), and HIF1A (NM_001243084.1) was predicted through the TargetScan 4.0 website (https://www.targetscan.org) and other online tools. Online databases and the sequences were evaluated using Snapgene software (http://snapgene.com). Interestingly, miR-561-3p showed sequence complementarity to the same region of the 3′-UTRs of *ZEB1, HIF1A,* and *MYC* genes.

###  Luciferase reporter assay

In the first step, the 3′-UTRs of *ZEB1, HIF1A,* and *MYC* genes were amplified using specific primers (Table 3). The highlighted sequences in Table 3 indicate the XhoI and NotI restriction sites within the designed primers. Pfu DNA polymerase (Fermentas, EP0501) was used for accurate amplification of the 3′-UTRs according to the manufacturer’s protocol. The PCR products and special scrambled sequence (AAGCTTCATAAGGCGCATAGC), as a negative control, were cloned into psiCHECK™-2 vector (Promega, C8021) immediately downstream of the stop codon of the renilla gene. Insertion of the PCR products into psiCHECK™-2 vector was confirmed by colony PCR, double digestion with NotI (Fermentas, ER0571) and Xho1 (Fermentas, ER0691) restriction enzymes, and sequencing (Niagen Noor Medical Genetics Laboratory, Tehran, Iran). Luciferase activity was measured with the Dual-Glo^®^ Luciferase Assay System (Promega, E2940) 48 h after transfection. The multiwell plate luminometer Renilla luciferase activity was normalized to that of firefly luciferase.

**Table 3:** Primer sequence for 3̀-UTRs PCR analysis (the XhoI and NotI restriction sites were highlighted in bold).

Gene	Sequence of oligonucelotides from 5′to 3′for 3′-UTRs	Product size
ZEB1	Forward: CCG**CTCGAG**GTAGTGAGCAAGTGTCTGAAGA Reverse: ATAAGAAT**GCGGCCGC**GGAAGACACCTAGAAATACACT	2631 bp
HIF1A	Forward: CCG**CTC GAG**AACCCTTCCAGAATTTTGCTTTA Reverse: ATAAGAAT**GCGGCCGC**TATGAGTTGGAGGTGTTGAAG	1500 bp
MYC	Forward: CCG**CTCGAG**AACTTGAACAGCTACGGAAC Reverse: ATAAGAAT**GCGGCCGC**AGTCAGAGTCAAAGAAAGTAAT	2033 bp

### MicroRNA transfection

miR-561-3p mimics and scrambled microRNA were obtained from GenePharma Co. (Shanghai, China). MDA‐MB‐231, BT-549 and MCF-7 cell lines were used as cells expressing PD-L1, and MCF-10 cell line was used as a negative control. The BC and negative control cell lines were transfected using HiPerFect Transfection Reagent (QIAGEN, Germany) according to the manufacturer’s protocol. Transfected cell lines were harvested 24, 48, and 72 h after transfection.

### Constitutive PD-L1 expression assay

The constitutive PD-L1 expression of different BC cell lines under standardized culture conditions was analyzed by flow cytometry. MCF‐7, MDA‐MB‐231, and BT-549 cell lines (1 × 10^5^ cells/well) were seeded into a 12-well plate, 24 h before transfection. BC cell line and MCF-10 cell line, as negative control, were transfected with miR-561-3p. After 48 h, the expression of cell-surface PD-L1 was investigated. In the first step, the cells were incubated with anti-CD16/32 (eBioscience, for mouse cells) to block the fc receptors. Then, the cells were stained with a LIVE/DEAD viability dye (Thermofisher Scientific) for 10 min at 4 °C in FACS buffer (PBS, 0.5% BSA, 2mM EDTA). In the next step, the cells were incubated with the PE anti-human CD274 (B7-H1, PD-L1) antibody (Biolegend, 329705) for 30 min at 4 °C and then analyzed with FlowJo 10.6.2 / 10.0.7 R2 software.

### Proliferation assay

Analysis of cell proliferation was performed using( 3-(4,5-dimethylthiazole-2-yl)-2, 5-biphenyl tetrazolium bromide (MTT) (Sigma, Schnelldorf, Germany). MDA-MB-231 and BT-549 cells (3 × 10^4^ cells/well) were seeded into a 96-well plate (Jet Biofil, Guangzhou, China). Then, the cells were transfected with miR-561-3p and scrambled microRNA. After 72 h, the cells were incubated with MTT (500μg/ml) for 4 h, then the supernatants were discarded and the insoluble formazan was dissolved in 100 µl dimethylsulphoxide (DMSO). The optical density was measured at 540 nm, and cell viability was defined relative to untreated control cells. All experiments were repeated three times, and the results were reported as mean ± SE.

### Apoptosis

MCF‐7, MDA‐MB‐231, and BT-549 cell lines (1 × 10^5^ cells/well) were seeded into a 12-well plate, 24h before transfection. BC cell lines were transfected with scrambled oligonucleotides and miR-561-3p using HiPerFect transfection reagent (QIAGEN. Germany), according to the manufacturer’s protocol. After 48 h, the cellular apoptotic rate was evaluated via Annexin V-FITC/PI staining kit (Biolegend. Austria) based on the provided instructions The results were analyzed using flow cytometry (FlowJo 10.6.2 / 10.0.7 R2 software). Each sample was run in triplicate.

### Cell cycle analysis

The cell cycle stage was analyzed by flow cytometry. MCF‐7, MDA‐MB‐231, and BT-549 cell lines (1 × 10^5^ cells/well) were seeded into a 12-well plate, 24 h before transfection. BC cell lines were transfected with miR-561-3p and scrambled microRNA. After 48 h, harvested cells were centrifuged, washed in phosphate-buffered saline (PBS), fixed in ice-cold 70% ethanol, and treated with 1 mg/ml RNase for 30 min. Then, 425µl Cell Staining Buffer (Biolegend 420201) and 25 µl Propidium Iodide Solution (Biolegend. 421301) were added and analyzed using FlowJo 10.6.2 / 10.0.7 R2 software. Each sample was run in triplicate.

### Statistical analysis data

RT‐qPCR results were analyzed using REST^®^, 2009 Software. All data were presented as mean ± SE. P values less than 0.05 were considered to be significant. Statistical analysis was performed using GraphPad Prism V.8 software. The t-test was used to compare cancerous and non-cancerous breast cells and samples. Differences between groups were analyzed by analysis of variance (ANOVA) when more than two groups were compared. Correlation analysis was performed using the Pearson test . 

### Ethics approval and consent to participate

All methods were carried out in accordance with relevant guidelines and regulations. All experimental protocols were approved by the Ethics Committee of Pasteur Institute of Iran (Ethical code: IR.PII.REC.1398.023). Informed consent was obtained to contribute, and to publish contributors' information from all subjects and/or their legal guardians.

### Informed consent

Written informed consent was acquired from all registered subjects.

### Supplementary Information

Supplementary Figure 1.

## Data Availability

All data generated or analyzed during this study are included in this published article.

## Abbreviations

*HIF1A*: Hypoxia-inducible factor 1-alpha
*ZEB1*: Zinc finger E-box-binding homeobox 1
*PD**-**L1*: Programmed cell death ligand 1
*PD**-**1*: Programmed cell death protein 1
BC: Breast cancer
miR-561: MicroRNA-561-3p
EMT: Epithelial-mesenchymal transition
CSC: Cancer stem cell
PI: Propidium iodide
