# Supplementary material for: MicroRNA-561-3p indirectly regulates the PD-L1 expression by targeting ZEB1, HIF1A, and MYC genes in breast cancer
Source: Sci Rep. 2024 Mar 10;14:5845. doi: 10.1038/s41598-024-56511-6 (PMC10925600; doi:10.1038/s41598-024-56511-6)
Supplement: Supplementary file 1 — Supplementary Figure 1. [file 41598_2024_56511_MOESM1_ESM.docx]

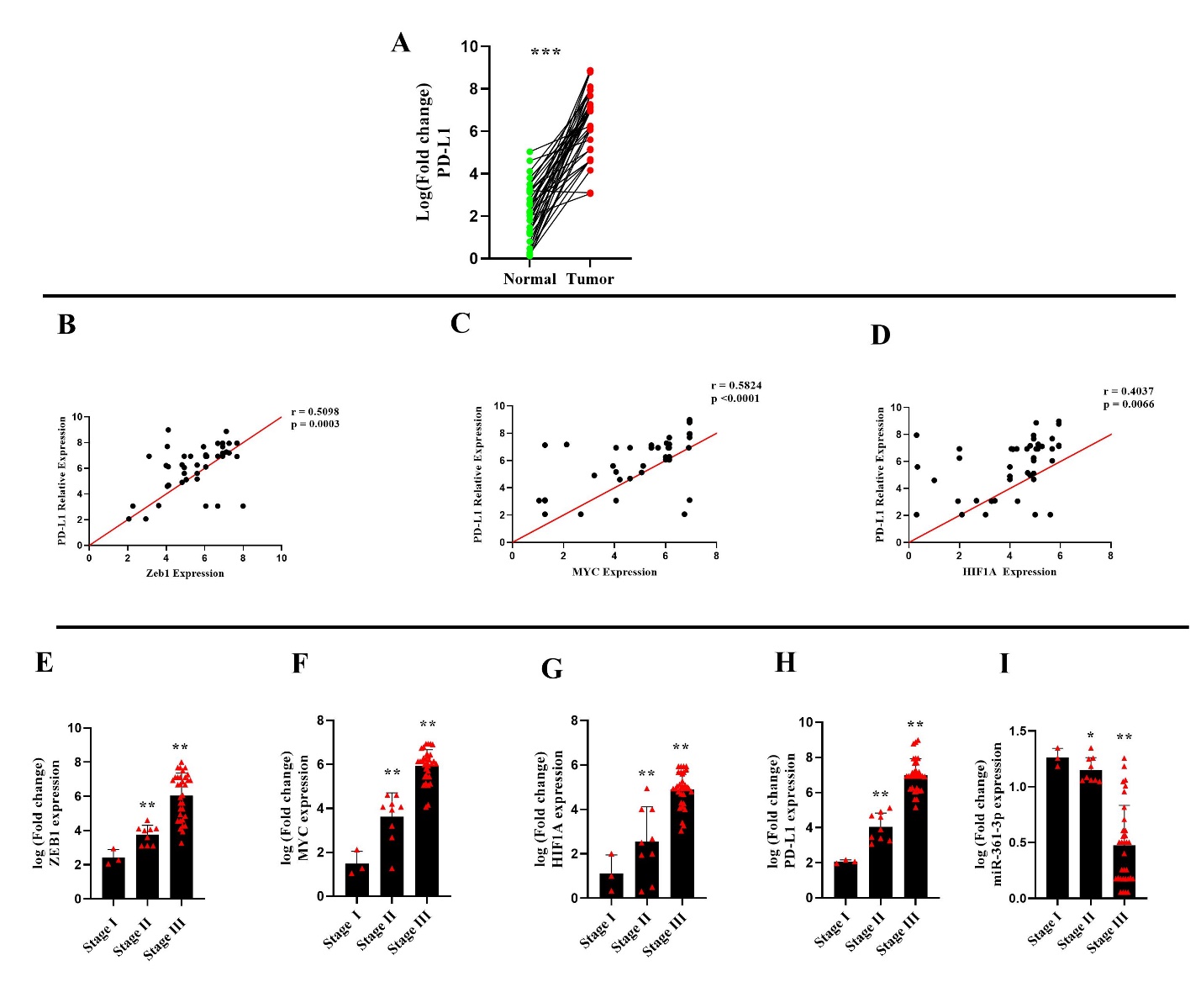


**SUPPLEMENTARY1:**

(A): the results showed that the expression of *PD-L1* gene was significantly increased in BC tissues compared with adjacent normal tissues (Supp.1A, P < 0.001).

(B, C, D): Pearson correlation analyses of *ZEB1*, *MYC*, and *HIF1A* gene expression in BC tissues demonstrated a positive correlation between *PD-L1* expression and *ZEB1* (Supp. 1B, r = 0.5098; P = 0.0003), *MYC* (Supp.1C, r = 0.5824; P < 0.0001), and *HIF1A* mRNA levels (Supp. 1D, r = 0.4037; P < 0.0066).

(E, F, G, H, I): The results of qRT-PCR revealed that the expression levels of *ZEB1* (Supp.1E, P < 0.001), *MYC* (Supp.1F, P < 0.001), and *HIF1A* (Supp.1G, P < 0.001), *PD-L1* genes (Supp.1H, P < 0.001) were significantly increased and miR-561-3p (Supp.1I, P < 0.001, P < 0.05) was abnormally down-regulated in BC tissues stage III compared with BC tissues stage I.
